# Bankart repair with remplissage vs. Latarjet procedure on recurrence, postoperative pain scores, external rotation, and Rowe score in patients with a Hill-Sachs lesion. A systematic review

**DOI:** 10.1016/j.xrrt.2023.08.001

**Published:** 2023-08-30

**Authors:** Casper L.J.H. Schrouff, Loek Verlaan

**Affiliations:** Department of Orthopaedic Surgery, CAPHRI School for Public Health and Primary Care, Maastricht University Medical Center, Maastricht, Netherlands

**Keywords:** Shoulder instability, remplissage, Latarjet, Hill-sachs lesion, Glenoid bone loss, anterior shoulder dislocation

## Abstract

**Background:**

Currently, recurrent anterior shoulder instability in patients with a Hill-Sachs lesion managed using the Bankart repair showed higher recurrent instability compared with the Latarjet technique. Addition of posterior capsulodesis with infraspinatus remplissage to the Bankart repair, known as Bankart with remplissage (BR), increases shoulder stability in patients with a Hill-Sachs lesion. BR can potentially match the low recurrence rates of the Latarjet procedure while being less invasive. This systematic review compares the Bankart repair with remplissage and Latarjet procedure on postoperative pain, external rotation range of motion, and recurrent instability in anterior shoulder instability patients with a Hill-Sachs lesion.

**Methods:**

A systematic search of the PubMed, Web of Science, and Cochrane Library databases was performed. Studies comparing BR and Latarjet on recurrent instability and/or visual analogue scale (VAS) pain score in anterior shoulder instability patients with a Hill-Sachs lesion were included. Expert opinion, conference presentations, editorials, abstracts, case reports, and nonclinical studies were excluded. Records were initially screened by title and abstract, during the second screening full text was consulted. Study quality was examined using the Methodological Index for Nonrandomized Studies criteria. Risk ratios were calculated for recurrent instability, and standardized mean difference (Cohen’s d) were calculated for VAS pain, external rotation, and Rowe score.

**Results:**

Eight of the 146 studies were included in the analysis. The study population consisted of a total of 845 patients, of whom 450 patients underwent the Latarjet procedure and 395 patients underwent BR. Three studies included revision surgery patients; more revision surgery patients were allocated to the Latarjet group. Risk ratios for recurrent instability varied from 0.45 to 2.41. Effect size varied for VAS pain from −2.28 to 0.04, for external rotation from −1.44 to 1.12, and for Rowe score from −0.67 to 1.37. Limitations of the included studies were differences in baseline demographics and functional outcomes.

**Conclusion:**

Recurrent instability seems equal between BR and Latarjet in patients with a Hill-Sachs lesion depth <10 mm. Latarjet showed superior external rotation than BR. Future research should examine patient demographics optimal for minimizing recurrent instability using BR.

The shoulder is the most mobile and most frequently dislocated joint,^25^ with an estimated incidence rate of 23.9 per 100,000 person-years.^41^ Shoulder dislocations occur 2.64 times more frequent in males relative to females.^41^ Additionally, a young age is a risk factor for shoulder dislocations, with almost half of the shoulder dislocations occurring in patients aged between 15 and 29 years.^41^

Over 95% of shoulder dislocations are anterior,^41^ which often result in subsequent anterior shoulder instability and recurrent dislocations.^26^ Anterior shoulder dislocations often result in avulsion of the labrum and bone from the anterior inferior glenoid, known as a Bankart lesion,^6^ and a dent posteriorly on the humeral head referred to as a Hill-Sachs lesion.^6^^,^^9^ The loss of bone from the anterior inferior glenoid results in a decreased articular area and reduced concavity for the humeral head, increasing the risk of recurrent dislocations.^34^ Anterior shoulder instability is amplified when a Hill-Sachs lesion is present.^10^^,^^34^ Anterior shoulder instability is associated with increased risk of osteoarthritis in the shoulder joint^27^ and over 50% of the patients with anterior shoulder instability will require surgical treatment.^20^^,^^21^^,^^26^

Surgical management of recurrent shoulder instability has demonstrated a lower incidence of arthropathy in comparison to conservative management.^21^ Patients with anterior inferior glenoid bone loss of less than 15% are usually managed with a Bankart repair.^3^^,^^34^ Patients with a Hill-Sachs lesion are commonly managed using the Latarjet technique,^36^ since the Bankart repair has shown to have a high failure rate in patients with Hill-Sachs lesions.^13^^,^^31^ Other factors that way in on the surgical management decision are age, degree of sport participation, type of sport, and laxity of the shoulder, which are incorporated in the instability severity index (ISI) score.^5^ All criteria on the ISI score are graded and the sum of these criteria have a maximum score of 10 points indicating the severity of shoulder instability.^5^

A different technique has been described by Purchase et al^36^ to manage Hill-Sachs lesions, where posterior capsulodesis and infraspinatus tenodesis is used to fill (remplissage) the Hill-Sachs lesion. Remplissage can be used in addition to a Bankart repair to increase shoulder stability in patients with a Hill-Sachs lesion.^36^ The Latarjet procedure and Bankart repair with remplissage (BR) have their disadvantages. The Latarjet has a relatively high complication rate compared to the Bankart repair, where graft complications such as non-union, fracture, and pain due to hardware failure are the most common complication.^23^ BR may limit internal-external rotation range of motion.^2^^,^^14^

A previous systematic review and meta-analysis by Haroun et al^16^ comparing BR and Latarjet procedure did not find a difference between postoperative pain, internal-external rotation range of motion, and dislocation recurrence. However, this systematic review and meta-analysis lacked evidence due to the limited quantity of available literature at the time.^16^ A different previous systematic review and meta-analysis by Gouveia et al^15^ comparing BR and bone block augmentation found similar functional outcomes. Meanwhile, BR may be associated with a greater recurrent instability rate than bone block augmentation for preoperative glenoid bone loss greater than 10%.^15^ However, the review included additional bone block augmentation surgery than the Latarjet procedure.^15^ Therefore, it does not allow to directly compare BR with the Latarjet on recurrence, external rotation range of motion, and postoperative visual analogue scale (VAS) pain scores.^15^ The most recent systematic review and meta-analysis by Davis et al^12^ compares BR to the Latarjet procedure and the Bankart repair combined as control group on return to sport, functional outcomes, and adverse events. Combining the outcomes of the Latarjet procedure and Bankart repair as one control group is inappropriate, since these surgeries reach significantly different outcomes.^28^ Therefore, false conclusions were made due to confounding of the outcomes reached with the Bankart repair, which is known to have greater recurrent instability rates than BR and the Latarjet procedure. Furthermore, the above mentioned meta-analyses^12^^,^^15^^,^^16^ did not account for important baseline characteristics in the analysis such as revision patients, glenoid bone loss, Hill-sachs lesion depth. This may have skewed the conclusions, favoring the BR, since patients with a greater risk of recurrent instability were included in the Latarjet group.^1^^,^^4^^,^^11^^,^^40^ Recent literature comparing BR and the Latarjet procedure in patients with a glenoid bone loss greater than 15% has shown similar recurrent instability rates.^18^ While, BR has shown less recurrent instability than the Latarjet procedure in a study with a mean glenoid bone loss below 15%.^19^ Contrastingly, the study of Hurley et al^22^ showed reduced recurrent instability in the Latarjet group compared to the BR group. These recent studies might bring new insights regarding clinical and patient reported outcomes. Therefore, this study aims to determine whether BR can match or decrease postoperative VAS pain, external rotation range of motion, and recurrent instability in anterior shoulder instability patients with a Hill-Sachs lesion when compared to the Latarjet technique.

## Methods

### Search strategy and protocol

This systematic review was conducted with reference to the Preferred Reporting Items for Systematic Reviews and Meta-Analyses guidelines.^32^ No protocol of this review has been registered. A search of the databases PubMed, Web of Science, and Cochrane library was performed for articles published before 10 March 2023 using the search terms Latarjet and remplissage. Additionally, to the search in the databases, backward and forward reference tracking has been performed on the included articles. Two investigators independently screened abstracts and performed a full-text review.

### Inclusion and exclusion criteria

Included studies had to compare BR with the Latarjet procedure and report postoperative VAS pain score and/or recurrence. Expert opinion, conference presentations, editorials, abstracts, case reports, and nonclinical studies were excluded. Only articles written in English or Dutch were included. Patients had to suffer from anterior shoulder instability with a Hill-Sachs lesion. Studies including only patients that needed revision surgery were excluded.

### Quality appraisal

The methodological quality of each selected study was assessed using the Methodological Index for Nonrandomized Studies.^39^ This is a 12-item list, assessing clarity of the stated study aim, inclusion of consecutive patients, data collection, appropriateness of the endpoints to the aim of the study, assessment of the study endpoints, follow-up period, loss to follow-up, prospective calculation of the study size, control group, contemporary groups, equivalence of the groups at baseline, and statistical analysis. Two reviewers assessed independently the quality of each study and awarded each item a not reported, reported but inadequately, adequately reported. Disagreements were discussed to reach consensus.

### Data extraction and abstraction

Data was extracted relating to shoulder stability measured by recurrent instability, which is defined as at least one postoperative episode of anterior subluxation or dislocation. In addition, data was collected regarding change in pain measured by VAS score, change in external rotation with the elbow at the side (ER1) measured in degrees, change in external rotation with the arm abducted at 90° (ER2) measured in degrees, and change in Rowe score, which assesses shoulder stability, motion, and function. Preoperative ISI score, Hill-Sachs lesion depth, and glenoid bone loss were extracted. In addition, data relating to the intervention was recorded: number of patients in the intervention and control group, number of males and females per group, population age, length of follow-up, mean instability episodes, number of patients receiving revision surgery, and postoperative comparative outcomes. One reviewer did the data extraction and abstraction.

### Synthesis

An effect size calculator (psychometrica.de) was used to calculate the effect sizes of the individual studies. Risk ratio for recurrent instability was calculated of the individual studies using (campbellcollaboration.org). Since continuous data from different scales were extracted, the standardized mean difference was calculated for effect size based on sample size (Cohen’s d with Hedges adjustment) for each study. The postoperative VAS pain score was subtracted from the preoperative VAS pain score, and the resulting effect size was calculated weighted on the sample size of each group using an effect size calculator.^35^ This was done to help with the interpretation of the standardized mean difference for the VAS pain score, since for all outcomes, a positive value for the standardized mean difference indicates a better result in the BR group. Effect sizes of 0.2 are usually considered small, those of 0.5 as moderate, and those of 0.8 as large.

## Results

### Study selection

**Figure 1:**
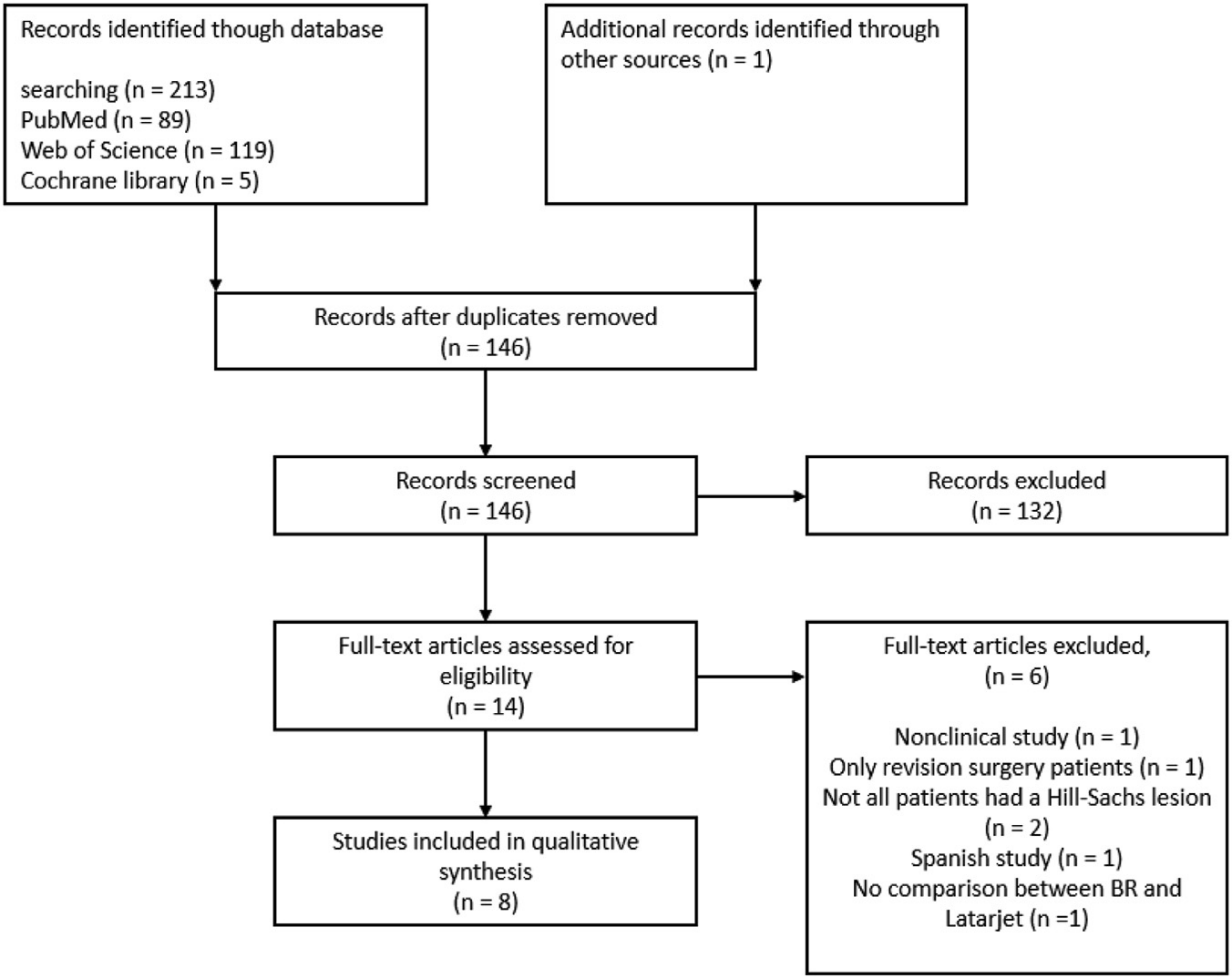
PRISMA flow diagram visualizing the article selection process. One study included BR and Latarjet groups but did not compare the groups on any outcome measure. *BR*, Bankart repair with Remplissage.

The study selection as shown in Figure 1 was performed by a single reviewer on PubMed using the aforementioned search string. A total of 146 articles were identified, after removing duplicates, through PubMed, Web of Science, and Cochrane Library, and one additional article was found using backward referencing. All articles were first screened based on title and abstract. Of the 146 articles, 14 articles passed the initial screening. Subsequently, articles were assessed based on their full text which provided eight articles to be included for the qualitative analysis. Two studies were excluded because not every patient had a Hill-Sachs lesion.^29^^,^^33^ One study was not a clinical trial.^24^ Other studies were excluded for Spanish language,^37^ inclusion of only revision surgery patients,^8^ not providing comparison between the BR and Latarjet group.^17^

### Study characteristics

The characteristics regarding study population, Hills-Sachs lesion depth, glenoid bone loss, ISI-score, follow-up length, and measured outcomes of the reviewed articles are presented in Table I. More male patients (79.5%) than female patients (20.5%) received shoulder surgery. Noteworthy was the study of Bah et al^4^ in which 81.4% of the BR group and 83.7% of the Latarjet group consisted of females. Average Hill-Sachs lesion depth and glenoid bone loss were 9.48 mm and 12.9% in the BR group and 8.42 mm and 14.8% in the Latarjet group, respectively. Two studies showed a significant difference in Hill-Sachs lesion depth^19^^,^^40^ and in glenoid bone loss^19^^,^^22^ between the BR group and the Latarjet group. Mean follow-up length varied across the studies from 24.7 to 74 months; follow-up length was not always similar between groups and not always separately reported for BR and Latarjet groups. All studies reported recurrence; while 4 studies^1^^,^^18^^,^^19^^,^^38^ only reported recurrent dislocations, the other studies^4^^,^^11^^,^^22^^,^^40^ reported recurrent instability. Preoperative ISI score was reported in 3 of the reviewed articles^4^^,^^11^^,^^40^ with a mean value of 4.4 in the BR group and a mean value of 4.7 in the Latarjet group. VAS pain score was reported in all studies; however, 3 studies^1^^,^^22^^,^^40^ reported only postoperative VAS pain score. In addition, one of these 3 studies reported only postoperative VAS pain score at one week.^1^ One study^22^ did not include a measurement of external rotation and one study^40^ only included a postoperative measurement of external rotation. Rowe score was reported in 3 of the included studies.^1^^,^^4^^,^^11^

**Table I:** Characteristics of the included studies.

Study	Study design (level of evidence)	Group	Patients (n)	Males/females (n/n)	Age (y)	Hills-Sachs lesion depth (mm)	Glenoid bone loss (%)	ISI-score	Instability episodes†/patients receiving revision surgery‡	Length of follow-up (mo)	Postoperative comparative outcomes
Bah et al 2018^4^	Retrospective comparative (III)	BR	43	8/35	24.3 (±6.5)	7.7 (±1.2)		4.2 (±1.8)	8 (±2.1)†	47.3 (±9.1)	SSV, Rowe score, Walch-Duplay score, patients with residual pain, VAS pain, ER1, ER2, Recurrence
		Latarjet	43	7/36	24.3 (±6.5)	7.9 (±1.5)		4.6 (±1.2)	10 (±3.5)†	47.3 (±9.1)	
Horinek et al 2022^19^	Retrospective comparative (III)	BR	70	60/10	26.7 (±9.3)	8.7 (±3.8)∗	12.3 (±10.9)∗			30 (not reported separately)	Return to same level of sport, patient satisfaction, recurrent dislocations, surgical complications, VAS pain, SANE, WOSI, ER1
		Latarjet	188	157/31	26.8 (±9.9)	3.9 (±2.6)∗	7.6 (±9.0)∗				
Horinek et al 2022^18^	Retrospective comparative (III)	BR	22	20/2	25.2 (±10.6)	9.3 (±3.9)	25.8 (±7.8)			28 (not reported separately)	Overall return to same level of sport, patient satisfaction, recurrent dislocations, surgical complications, hematoma or infection
		Latarjet	25	21/4	29.6 (±14.0)	5.6 (±4.4)	25.1 (±9.0)				
Hurley et al 2022^22^	Retrospective comparative (III)	BR	41	28/13	29.6 (±10.8)		7.3 (±7.8)∗		2‡^,^∗	58.5 (±24)	WOSI score, VAS pain, VAS sport, SSV, SIRSI score, satisfaction, recurrence
		Latarjet	26	21/5	32.3 (±12.7)		19.1 (±4.7)∗		11‡^,^∗	52 (±23.4)	
Saremi et al 2021^38^	Retrospective comparative (III)	BR	68		27 (not reported separately)					74 (not reported separately)	ER1, ER2, VAS pain,
		Latarjet	26								
Abouelsoud and Abdelrahmen 2015^1^	Prospective comparative (II)	BR	16	15/1	28.2 (not reported separately)				>3	31 (not reported separately)	Rowe score, ER2, VAS pain (1 week post-op)
		Latarjet	16	14/2					>3		
Cho et al 2016^11^	Retrospective comparative (III)	BR	37	34/3	24.8 (±9.0)	6.8 (±1.7)	8.5 (±5.8)	4.2 (±1.0)	10‡^,^∗	24.7 (±9.5)∗	Apprehension, VAS rest/motion, Rowe score, UCLA score, ER1, ER2, muscle strength
		Latarjet	35	32/3	27.8 (±7.9)	6.4 (±2.4)	9.8 (±6.1)	4.5 (±1.4)	22‡^,^∗	30.4 (±11.2)∗	
Yang et al 2018^40^	Retrospective comparative (III)	BR	98	94/4	28.3 (±10.0)	14.9 (±7.1)∗	10.4 (±6.8)	4.8 (±1.9)	23‡^,^∗	38 (not reported separatedly)	WOSI, SANE, VAS pain, ER1, ER2, recurrence, complications
		Latarjet	91	86/5	30.0 (±12.1)	18.3 (±3.2)∗	12.3 (±8.7)	4.9 (±1.3	39‡^,^∗		

*BR*, Bankart repair with remplissage; *SSV*, subjective shoulder value; *VAS*, Visual Analogue Scale; *ER1*, external rotation with elbow by the side; *ER2*, external rotation with the arm abducted at 90°; *WOSI*, Western Ontario shoulder instability; *SIRSI*, shoulder instability-return to sport after injury; *SANE*, single assessment numeric evaluation; *ISI-score*, Instability Severity Index Score.

∗ Significant preoperative difference between Bankart repair with Remplissage group and Latarjet.

† Mean number of pre-operative instability episodes.

‡ Number of patients receiving revision surgery.

### Outcome data and effect sizes

The results of the data analysis are shown in Table II. No study showed a significant difference in recurrent instability between the BR group and the Latarjet group. Three studies^4^^,^^11^^,^^19^ showed a reduced risk ratio of 0.45 (0.05; 3.65), 0.80 (0.23; 2.78), and 0.95 (0.14; 6.35) for recurrent instability in the BR group. While, two studies^22^^,^^40^ show an increased risk ratio of 1.59 (0.33; 7.56) and 2.41 (0.90; 6.50) for recurrent instability in the BR group. One study showed a significant decrease in VAS pain score for the BR group compared to the Latarjet group.^19^ A different study showed a significantly lower postoperative VAS pain score in the Latarjet group than the BR group.^40^ VAS pain effect sizes showed one large effect size for the Latarjet group (d = −2.28)^4^ and one small effect size for the BR group (d = 0.36).^19^ Three of the five studies that reported changes in ER1 showed larger decreases in range of motion in the BR group than the Latarjet group. Standardized mean differences of these studies showed two moderate effect sizes (d = −0.51,^4^ d = −0.76^18^) and one large effect size (d = −1.44).^19^ While, one of the five studies showed a smaller decrease in ER1 range of motion in the BR group, with a large effect size (d = 1.12).^38^ Changes in ER2 were reported in 4 studies, where one study did not report standard deviations.^1^ Therefore, the standardized mean difference could only be calculated for three studies. One study showed a smaller decrease in ER2 in the Latarjet group with a large effect size (d = −1.12).^4^ Two studies showed a smaller decrease in ER2 in the BR group, where one study had a small effect size (d = 0.22)^11^ and one study a moderate effect size (d = 0.63).^38^ Changes in Rowe score were reported in 3 studies, where one study showed no difference (d = 0.04),^11^ one study showed a greater increase with moderate effect size in the Latarjet group (d =−0.67),^4^ and one study a greater increase with large effect in the BR group (d = 1.37).^1^

**Table II:** Effect of Bankart repair with remplissage compared to Latarjet on recurrent instability, VAS pain, external rotation, and Rowe score.

Study	Group	Recurrent instability (n)	Risk ratio recurrent instability BR/Latarjet	Change in VAS pain score	Standardized mean difference of VAS pain∗	Change in ER1 (°)	Change in ER2 (°)	Standardized mean difference of ER1/ER2	Change in Rowe score	Standardized mean difference of Rowe score
Bah et al 2018^4^	BR (n = 43)	4 (9%)	0.80 (0.23; 2.78)	−2.0	−2.28	−10.1	−13.2	−0.51/−1.12	43.8	−0.67
	Latarjet (n = 43)	5 (11%)		−3.2		−3,7	−0.3		50.3	
Horinek et al 2022^19^	BR (n = 70)	1 (1.4%)	0.45 (0.05; 3.65)	−1.9	0.36	−4.0	-	−1.44/-	-	-
	Latarjet (n = 188)	6 (3.6%)		−0.9		19.0	-		-	
Horinek et al 2022^18^	BR (n = 22)	0	-	−2.0	0.04	−4.0	-	−0.76/-	-	-
	Latarjet (n = 25)	0		−1.9		10.0	-		-	
Hurley et al 2022^22^	BR (n = 41)	5 (12.2%)	1.59 (0.33; 7.56)	-	-	-	-	-	-	-
	Latarjet (n = 26)	2 (7.7%)		-		-	-		-	
Saremi et al 2021^38^	BR (n = 68)	1 (1.5%)	-	−7.9	-	−7.0	−10.4	1.12/0.63	-	-
	Latarjet (n = 26)	0		−6,3		−18.5	−16.6		-	
Abouelsoud and Abdelrahmen 2015^1^	BR (n = 16)	0	-	-	-	-	−10.0	−/−	60.5	1.37
	Latarjet (n = 16)	1 (6.3%)		-		-	−15.0		55.9	
Cho et al 2016^11^	BR (n = 37)	2 (5.4%)	0.95 (0.14; 6.35)	−0.8	−0.13	−8.0	−4	0.12/0.22	47	0.04
	Latarjet (n = 35)	2 (5.7%)		−1.3		−10	−7		50	
Yang et al 2018^40^	BR (n = 98)	13 (13.3%)	2.41 (0.90; 6.50)							
	Latarjet (n = 91)	5 (5.5%)								

*BR*, Bankart repair with remplissage; *VAS*, Visual Analogue Scale; *ER1*, external rotation with elbow by the side; *ER2*, external rotation with the arm abducted at 90°.

∗ Pre- and postoperative score were inverted in the calculator when calculating the standardized mean difference.

### Quality appraisal

The results of the study quality appraisal using the Methodological Index for Nonrandomized Studies criteria are shown in Table III. All studies scored high for the follow-up period and control group, since all studies had a follow-up period longer than 24 months. Two years or longer is an adequate follow-up duration, since shoulder rehabilitation after surgery is completed in 4 to 6 months.^7^ All studies failed to mention whether a protocol was used for the prospective data collection. Only one study reported a prospective power calculation of the study size.^40^ Outcome measures were not reported in the study aim in all studies. Blind evaluation of objective outcomes was reported in one study.^4^ In addition, this case-control study^4^ managed to have equal baseline group characteristics (see Table I for a global indication). Three studies had a loss to follow-up of more than 5%, while the proportion lost to follow-up did exceed the proportion experiencing the major endpoint (recurrent instability).^4^^,^^22^^,^^40^ Lastly, only three studies used adequate statistical analyses to answer the study aim.^18^^,^^19^^,^^40^

**Table III:** Risk of bias assessment of the included studies using the Methodological Index for Nonrandomized Studies.

Study	A clearly stated aim	Inclusion of consecutive patients	Prospective collection of data	Endpoints appropriate to the aim of the study	Unbiased assessment of the study endpoint	Follow-up period appropriate to the aim of the study	Loss to follow up less than 5%	Prospective calculation of the study size	An adequate control group	Contemporary groups	Baseline equivalence of groups	Adequate statistical analyses	Total
Bah et al 2018^4^	1	2	0	2	1	2	0	0	2	2	2	0	14
Horinek et al 2022^19^	1	2	0	2	0	2	2	0	2	2	0	2	15
Horinek et al 2022^18^	1	2	0	2	0	2	2	0	2	2	1	2	16
Hurley et al 2022^22^	1	2	0	1	0	2	0	0	2	2	0	1	11
Saremi et al 2021^38^	1	2	0	1	0	2	2	0	2	2	0	0	12
Abouelsoud and Abdelrahmen 2015^1^	1	2	0	0	0	2	2	0	2	2	0	0	11
Cho et al 2016^11^	1	2	0	1	0	2	2	0	2	0	1	1	12
Yang et al 2018^40^	1	2	0	2	0	2	0	2	2	2	0	2	15

Scores: 0 = not reported, 1 = reported but inadequately, 2 = adequately reported.

## Discussion

### Summary of the main findings

The purpose of this article was to provide a review of the current literature for consensus on the potential benefit of BR when compared to Latarjet in anterior shoulder instability patients with a Hill-Sachs lesion. It was found that BR and Latarjet perform similar on recurrent instability over a period of two to four years in patients with a Hill-Sachs lesion. With exception of one study with a large effect size (d = −2.28),^4^ BR and Latarjet performed similar on long-term postoperative VAS pain score. Latarjet performed better than BR at preserving and even in two studies^18^^,^^19^ increasing postoperative ER1. However, post-operative ER2 results were inconclusive between surgeries, where in one study a large effect size (d = −1.12)^4^ favoring Latarjet and two studies with a moderate^38^ and a small^11^ effect size favoring BR (d = 0.63, d = 0.22) were observed. Lastly, no clear difference was observed in Rowe score between BR and Latarjet, with one study showing no difference (d = 0.04),^11^ one study showing a large effect size in favor of BR (d = 1.37),^1^ and one study showing a moderate effect size in favor of Latarjet (d = −0.67).^4^

### Quality of the evidence

One large risk ratio was calculated for recurrent instability in the BR group (RR = 2.41), which indicates that patients in the BR group have a greater risk of recurrent instability than patients in the Latarjet group. When comparing preoperative characteristics in this study compared to other included studies in this review, two differences could be observed in the patient characteristics.^40^ First, patients had a greater Hill-Sachs depth and width in both groups.^40^ The patients in the Latarjet group had even a significantly greater Hill-Sachs depth than patients in the BR group,^40^ indicating that Latarjet is a preferable option in patients with a greater Hill-Sachs lesion. Second, this study included patients that needed revision surgery for anterior shoulder instability, 23 of the 94 patients in the BR group and 39 of the 86 patients in the Latarjet group.^40^ Currently, Latarjet is the preferred procedure to address recurrent postoperative shoulder instability. The Latarjet group in this study had significantly more revision patients with a significantly greater number of previous surgeries for revision patients.^40^ The study of Bah et al^4^ is a case-control study, and baseline demographic characteristics were similar for both groups; therefore, this study provides a better comparison. The study of Cho et al^11^ does not have all baseline characteristics similar between the BR and Latarjet group. For instance, there are more patients with previous surgery in the Latarjet group (n = 22/35) than in the BR group (n = 10/37).^11^ Results of a revision surgery are usually inferior to the results of a primary stabilization surgery.^30^ Therefore, the results may be skewed in favor of the BR procedure, since less revision surgery patients were present in the BR group.^11^

With exception of one study,^4^ BR and Latarjet were equally capable in reducing VAS pain score for patients. In the study of Bah et al^4^ were preoperative VAS pain scores higher in the Latarjet group (VAS pain = 6.01) compared to the BR group (VAS pain = 5.02). Postoperative VAS pain scores were similar in both groups at last follow-up with a VAS pain score of 2.8 in the Latarjet group and a VAS pain score of 3.04 in the BR group.^4^

With exception of one study,^38^ the included articles saw a better preserved ER1 using the Latarjet. However, the study of Saremi et al^38^ did not provide pre and post ER1 and ER2 results but only reported the change in ER1 and ER2 for both groups. Moreover, very few baseline demographic characteristics were reported for both groups, making it difficult to place the results of this study in perspective.^38^ Only three studies could be used to calculate effect sizes for ER2, of which one did not report pre- and post-results.^38^ One large effect size for ER2 was calculated (d = −1.12) for Latarjet group^4^ and one small effect size was found in favor of the BR group.^11^ For both studies, similar preoperative ER2 were observed for the BR and Latarjet groups.^4^^,^^11^

Rowe score was reported in three studies,^1^^,^^4^^,^^11^ with inconclusive results across these studies. One large effect size for Rowe score was calculated for the BR group (d = 1.37).^1^ However, there were no baseline demographics and description of treatment selection reported.^1^ Contrastingly, a moderate effect size for Rowe score was calculated for the Latarjet group (d = −0.67).^4^ In this study, baseline demographic data and Rowe score were similar between the BR and Latarjet group.^4^

### Applicability of the evidence

Due to the similar long-term results, BR seems to be an adequate treatment option for patients with anterior shoulder instability. However, due to the risk ratio of 2.41 in patients with a large Hill-Sachs lesion and glenoid bone defect,^40^ BR may better not be used in this population. According to the demographics of the included reviewed articles, the cut-off for using BR seems to be a Hill-Sachs lesion depth of ∼10 mm. Future research could investigate save cut-off values for glenoid bone, ISI-score, Hill-Sachs lesion depth and width when using BR. Secondly, BR could possibly reduce external rotation to a greater extent than the Latarjet procedure. Therefore, based on the outcomes reported in this review, BR seems to be a slightly inferior treatment for anterior shoulder instability in patients with a Hill-Sachs lesion than the Latarjet procedure. However, the conclusions of this review are only based on recurrent instability, long-term VAS pain, external rotation, and Rowe score. Therefore, implementation of the results from this review should be done with careful consideration. For instance, BR yields fewer complications than the Latarjet procedure,^19^ which is proposed as the main benefit of BR over Latarjet. However, little literature is available comparing complications between BR and Latarjet. Future randomized control trails could identify whether BR may be a valuable treatment option over the Latarjet procedure in specific populations. Long-term VAS pain scores were similar for BR and Latarjet. However, BR is a less invasive surgery than Latarjet and might therefore see an earlier postoperative reduction in VAS pain score. Future research could compare early postoperative pain scores using BR and Latarjet.

### Limitations

The main limitation of this review was the lack of available randomized control trails. All included studies suffered from some methodological weaknesses. For instance, unequal baseline demographics and functional outcomes between treatment groups, which may introduce confounding factors. The choice for BR or Latarjet was made by the surgeon in all reviewed articles, which may influence the results. Currently, the open Latarjet procedure has been known as the best treatment option for recurrent anterior shoulder instability and is the more common choice for revision surgery due to reduced recurrent instability rates.^18^ Therefore, it is likely that patients that were expected to have a greater chance of recurrent instability were treated using the Latarjet procedure. Another limitation is the lack of ISI scores reported in the reviewed articles, since this score is often used for treatment decision making. Additionally, no information was provided whether the Hill-Sachs lesion was an on-track or off-track lesion, which is important for surgical decision making, since off-track lesions have a high recurrent instability rate after arthroscopic Bankart repair compared to on-track lesions.^17^ Statistical analyses were in the majority of the reviewed articles not appropriate (see Table III), while some studies did not compare functional outcomes between groups or failed to report the used statistical analysis. Another major limitation of this review was that one assessor executed the data extraction, abstraction, and synthesis of the review process.

## Conclusion

This systematic review identified eight studies that compared BR to Latarjet in anterior shoulder instability patients with a Hill-Sachs lesion. BR and Latarjet seem to perform similar on recurrent instability in patients with a Hill-Sachs lesion depth <10 mm. Future research could examine cut-off values for patient demographics such as Hill-Sachs lesion depth and width, glenoid bone score, ISI score, to minimize recurrent instability using BR. Long-term postoperative VAS pain scores were equally reduced for BR and Latarjet. Future studies could examine early postoperative pain using BR compared to Latarjet. Latarjet seems to preserve a greater external rotation range of motion than BR. Lastly, no clear difference could be observed in Rowe score between BR and Latarjet.

## Disclaimers:

Funding: No funding was disclosed by the authors.

Conflicts of interest: The authors, their immediate families, and any research foundation with which they are affiliated have not received any financial payments or other benefits from any commercial entity related to the subject of this article.
